# Retrophylogenomics in rorquals indicate large ancestral population sizes and a rapid radiation

**DOI:** 10.1186/s13100-018-0143-2

**Published:** 2019-01-21

**Authors:** Fritjof Lammers, Moritz Blumer, Cornelia Rücklé, Maria A. Nilsson

**Affiliations:** 1 Senckenberg Biodiversity and Climate Research Centre, Senckenberg Gesellschaft für Naturforschung, Senckenberganlage 25, 60325 Frankfurt am Main, Germany; 2 LOEWE Centre for Translational Biodiversity Genomics (LOEWE-TBG), Senckenberganlage 25, 60325 Frankfurt am Main, Germany; 3 Institute for Ecology, Evolution and Diversity, Goethe University Frankfurt, Biologicum, Max-von-Laue-Straße 13, 60439 Frankfurt am Main, Germany

**Keywords:** Evolution, Phylogenetics, Whales, Transposable elements, Retrotransposon

## Abstract

**Background:**

Baleen whales (Mysticeti) are the largest animals on earth and their evolutionary history has been studied in detail, but some relationships still remain contentious. In particular, reconstructing the phylogenetic position of the gray whales (Eschrichtiidae) has been complicated by evolutionary processes such as gene flow and incomplete lineage sorting (ILS). Here, whole-genome sequencing data of the extant baleen whale radiation allowed us to identify transposable element (TE) insertions in order to perform phylogenomic analyses and measure germline insertion rates of TEs. Baleen whales exhibit the slowest nucleotide substitution rate among mammals, hence we additionally examined the evolutionary insertion rates of TE insertions across the genomes.

**Results:**

In eleven whole-genome sequences representing the extant radiation of baleen whales, we identified 91,859 CHR-SINE insertions that were used to reconstruct the phylogeny with different approaches as well as perform evolutionary network analyses and a quantification of conflicting phylogenetic signals. Our results indicate that the radiation of rorquals and gray whales might not be bifurcating. The morphologically derived gray whales are placed inside the rorqual group, as the sister-species to humpback and fin whales. Detailed investigation of TE insertion rates confirm that a mutational slow down in the whale lineage is present but less pronounced for TEs than for nucleotide substitutions.

**Conclusions:**

Whole genome sequencing based detection of TE insertions showed that the speciation processes in baleen whales represent a rapid radiation. Large genome-scale TE data sets in addition allow to understand retrotransposition rates in non-model organisms and show the potential for TE calling methods to study the evolutionary history of species.

**Electronic supplementary material:**

The online version of this article (10.1186/s13100-018-0143-2) contains supplementary material, which is available to authorized users.

## Background

The bifurcating tree of life, where at each speciation event one ancestral lineage split into two new species, is a concept deeply rooted in the field of evolutionary biology. The opposite, that several new lineages diverge from the same speciation event, a so called polytomy, is mostly regarded as an artefact of limited phylogenetic information [1]. The sequencing and analyses of complete genomes was expected to finally resolve ambiguous relationships by providing enormous amounts of data [2]. Instead of resolving long standing phylogenetic controversies, genome-scale datasets revealed a lot of natural complexity in the phylogenetic data that previously had been deemed as noise [3, 4].

The evolutionary history of baleen whales (Mysticeti) is a prominent example of a phylogeny that lacked a scientific consensus for a long time [5–8]. In particular, the relationships among rorquals (Balaenopteridae) and gray whales (Eschrichtiidae) were contentious. While some studies showed that the only extant species of gray whales (*Eschrichtius robustus*) is phylogenetically placed within rorquals [6–8], others placed the gray whale as a sister group to rorquals, which was expected given its different morphology and feeding behaviour [5, 9]. Recently, whole-genome sequencing (WGS) of nearly all extant baleen whale species suggested that the rapid radiation of rorquals might represent a hard polytomy [10]. To further explore if the baleen whale phylogeny contains a polytomy, we use transposable element (TE) insertions. TEs are a robust and independent type of phylogenetic markers, that overcomes many limitations of sequence based phylogenetics, i.e. based on single nucleotide variants (SNV) [11]. Furthermore, TEs evolve neutrally and occur interspersed throughout the genome. Hence, they avoid potentially biased phylogenetic signals from gene tree error or linkage disequilibrium that can occur in sequence-based multi-locus analyses [12]. In addition, TE insertions are virtually homoplasy-free because parallel insertions in the large genomic space are very rare [11]. Also, they are less prone to reversals or mutational saturation that can affect SNV-based phylogenetic inference [11].

In baleen whale genomes, the most abundant TEs are short and long interspersed nuclear elements (SINEs and LINEs), covering 24.5% of the bowhead whale genome [10, 13]. The most abundant SINE family in baleen whales are CHR2 elements, which are named after their presence in **C**etacea, **H**ippopotamidae and **R**uminants [14] and emerged at least 56 million years ago (Mya). Like most other SINEs, the non-autonomous CHR2 elements are derived from a tRNA sequence. They are mobilized by the enzymatic machinery of LINE1 elements via an RNA intermediate that is reverse transcribed to cDNA and reintegrated into the genome. Compared to LINEs, their relatively high insertion frequencies make SINEs ideally suited for phylogenetic inference in mammalian genomes [11]. TEs have a long history of being used as phylogenetic markers for different cetacean groups [15–17].

Due to advances in genome sequencing and software development thousands of TE insertions can be inferred from multiple genomes across species and individuals [18, 19]. Thus, genome-scale TE detection was successfully applied to analyze retrotransposition in several vertebrate clades outside humans [20–23]. Furthermore, WGS based approaches proved extremely valuable in phylogenetic inference because they can increase the number of discovered TE insertions a thousand-fold, providing enhanced statistical power and the possibility to detect processes of reticulate evolution [23]. By contrast, PCR-based approaches have relied on tedious and time-consuming experimental work to find a few dozens of phylogenetically informative TE insertions from hundreds to thousands of candidate loci [24, 25]. Selection of candidate loci using an experimental approach was often based on a single genome sequence, introducing an ascertainment bias in the phylogenetic signal [17, 26, 27] that can be avoided by the use of large scale WGS sequencing and bioinformatic pipelines.

Here, we identified 91,859 CHR2 insertions in the available baleen whale genomes. This dataset was used to reconstruct the rorqual species tree and allowed us to quantify evolutionary conflict originating from their rapid radiation that took place approximately 8 Mya, coinciding with the onset of modern global oceanic circulation.

## Results

### WGS mapping and TE variation discovery

We mapped 11 WGS datasets from baleen whales with a coverage depth between 7 and 30 X to the bowhead whale (*Balaena mysticetus*) genome sequence [13] (Additional file 1: Table S1). From the mapped data, the Mobile Element Locator Tool (MELT) [19] called 488,373 non-reference (i.e. absent from the bowhead whale genome) CHR2 insertions, of which 327,488 (67.1%) passed stringent quality filtering. The bowhead whale is a natural outgroup to rorquals and gray whales, hence we focused on calling non-reference insertions in the 11 baleen whales to obtain an ascertainment bias free marker set for rorquals and gray whales. The total number of extracted CHR2 insertion calls per species ranged between 27,994 and 38,182, except for the North Atlantic right whale (*Eubaleana glacialis*), for which 6608 were found (Table 1). The North Atlantic right whale diverged from the bowhead whale about 4.4 Mya, hence fewer variable CHR2 loci reflect a closer genetic distance. In comparison, the divergence time of right whales and the bowhead whale to rorquals and gray whales is ~ 28 Ma. For clarity, we follow the nomenclature by ref. 10 to include the gray whale within rorquals sensu lato (Balaenopteridae + Eschrichtiidae).

**Table 1:** Numbers of all CHR2 insertion calls, as well as the amount of heterozygous insertions (Het) in baleen whale genomes compared to the bowhead whale genome

Sample	No CHR2 calls	Het
Blue whale	37,133	26,942
Fin whale	27,994	13,712
Gray whale (eastern) A	36,064	14,648
Gray whale (eastern) B	38,182	17,449
Gray whale (western) A	32,057	24,922
Gray whale (western) B	32,735	22,544
Humpback whale	28,618	14,622
Minke whale	28,606	12,089
North Atlantic right whale	6608	4221
Sei whale A	29,874	11,242
Sei whale B	29,617	11,079
Total	327,488	173,470

Extensive simulations to test the performance of MELT on our dataset showed that a sequencing depth of 5 X or higher is sufficient to reach true positive rates (TPR) of 99% for CHR2 insertions (Additional file 1: Figure S1A). Similarly, 92% of called CHR2 insertions were correctly recognized as homozygous indicating a high genotype accuracy on our dataset (Additional file 1: Figure S1B). MELTs internal filtering reduced sensitivity slightly (Additional file 1: Figure S1C, D), however, our simulations showed that the most effective filters affected all mapped genomes equally because they were based on properties of the reference genome, e.g. the presence of low-complexity regions (Additional file 1: Figure S2). Hence, these filters are not expected to create biases between samples that would influence phylogenetic inference. Furthermore, MELT-Split, which jointly genotypes all genomes, highly improved the detection of orthologous insertions compared to analyzing each genome individually and later combining the results. In summary, the simulations showed that our approach generated a dataset of high-quality baleen whale TE insertions with the corresponding orthology information that are suitable for evolutionary analyses.

### TE phylogenomics recovers rorqual speciation history

**Fig. 1:**
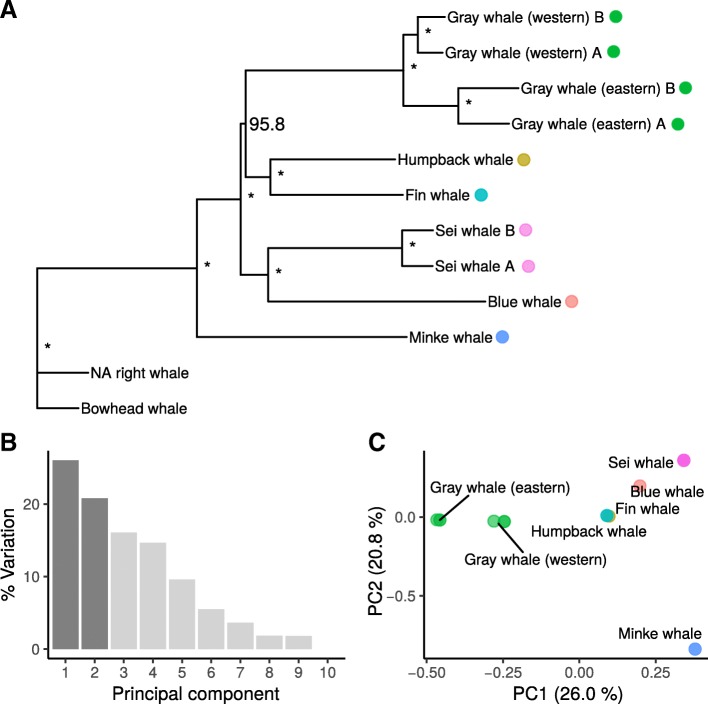
Phylogenetic signal calculated from 91,859 CHR2 insertions in baleen whales. **a** Neighbor-Joining tree based on CHR2 insertions. All nodes received bootstrap values of 95% or higher (100% shown as asterisk). **b** Percentage of variation explained by principal components 1–10 in the PCA. **c** Scatterplot of the first two principal components (PC1 and PC2) among baleen whale genomes

By creating a presence-absence matrix from 327,488 genotyped CHR2 insertion sites in all genomes, 91,859 orthologous integration events were identified that took place during the evolution of baleen whales. Based on the presence-absence matrix, phylogenetic trees were reconstructed  using Dollo parsimony, Bayesian inference (BI), and Neighbor-Joining (NJ) methods. The three reconstruction methods indicated a common monophyletic origin of Balaenopteridae and Eschrichtiidae (Fig. 1a, Additional file 1: Figure S3) and placed the gray whale as the sister species to the fin whale (*Balaenoptera physalus*) and humpback whale (*Megaptera novaeangliae*) clade. The minke whale (*Balaenoptera acutorostrata*) was reconstructed as the most basal rorqual species. In the NJ and BI trees, blue whale (*Balaenoptera musculus*) and sei whales (*Balaenoptera borealis*) formed a monophyletic clade as a sister group to the fin, humpback and gray whales. The CHR2 Dollo parsimony tree differed slightly from this topology because it reconstructed blue and sei whale as two separate lineages outside the fin, humpback and gray whale clade (Additional file 1: Figure S3 A). All trees received high node support with bootstrap values > 0.95 (Dollo parsimony, NJ) and 100% posterior probabilities (BI).

Although these tree reconstruction methods can by design only yield bifurcating topologies and cannot take conflicting genomic signals into account, considerable amount of phylogenetic conflict is indicated by low consistency indices (CI) (ranging between 0.629 and 0.646). The CI is a measure for tree support that indicates the fraction of minimum character changes compared to the observed number of changes, i.e. the tree length. If all character changes are consistent with the reconstructed tree, the CI is 1.0.

Analyzing the phylogenetic signal from CHR2 insertions among rorquals sensu lato using a principal component analysis (PCA) resulted in only the minke whale being clearly separated from the other species in the first two components, which together explained more than 50% of the variance in the dataset (Fig. 1b and c). While most species were found to be distinct along the first component, gray, fin and humpback whale were nearly indistinguishable on the second component. Furthermore, on the second component, the intraspecific differentiation between the two gray whale populations was as high as between other species pairs (Fig. 1c).

### Network analysis reveals phylogenetic conflict

**Fig. 2:**
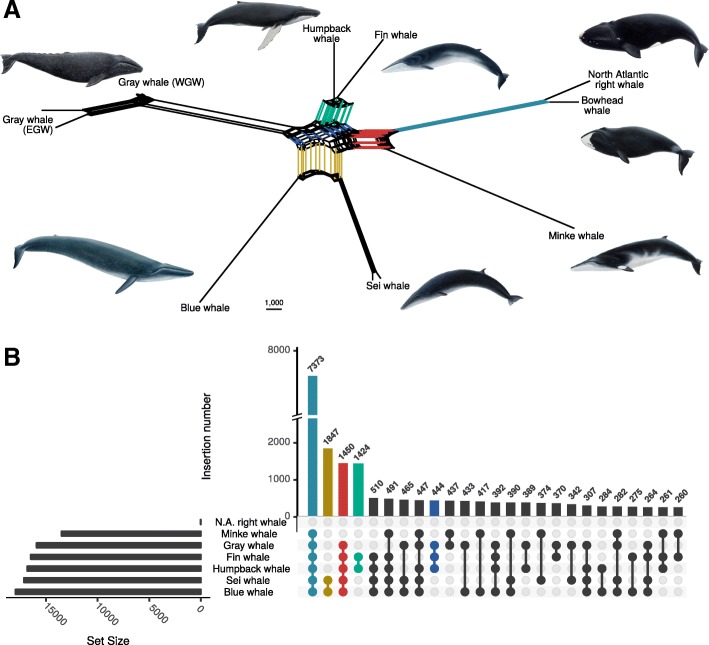
Phylogenetic conflict among baleen whales inferred from CHR2 insertions. **a** Phylogenetic median-joining network based on 91,859 CHR2 insertions. **b** Distribution of phylogenetic signals in the dataset. Each synapomorphic CHR2 insertion is considered a phylogenetic signal for the common ancestry for the taxa carrying the insertions. The x-axis shows synapomorphic CHR2 insertions between species listed on the left-hand side. Bars on the y-axis show the number of insertions for the respective synapomorphies. The set sizes on the left-hand side show the total number of insertions present per species. Whale paintings are by Jon Baldur Hildberg (www.fauna.is)

The low CIs of the phylogenetic trees indicate considerable amounts of phylogenetic conflict in the baleen whale genomes. To further explore these evolutionary signals, a median-joining network was calculated in order to uncover signals that otherwise remain hidden by traditional bifurcating tree-reconstruction algorithms. The phylogenetic network of CHR2 insertions showed a star-like web in the center of Balaenoptera and Eschrichtiidae (rorquals sensu lato) (Fig. 2a). Edges in the network that cluster the gray whale with either the blue and sei whales and/or fin and humpback whales had similar lengths, thus indicating equally strong phylogenetic signal for both topologies.

A quantification of shared CHR2 insertions in baleen whales showed that the four strongest phylogenetic signals support the NJ tree (Fig. 2b) and are in agreement with the evolutionary history of rorquals inferred from genomic sequence analyses [10]. For example, the strongest signal consisted of 7373 synapomorphic CHR2 insertions shared by all rorquals sensu lato and supports a common ancestry of this clade. Within rorquals, 1450 insertions support that the gray whale diverged after the minke whale, confirming the paraphyly of rorquals sensu stricto. The monophyly of blue and sei whale as well as of fin and humpback whale was supported by 1847 and 1424 insertions, respectively. These strong signals match the well supported nodes in the reconstructed phylogenetic tree (Fig. 1a): the minke whale is clearly distinct from the other rorquals, and the sister group relationships of blue and sei whale as well as of fin and humpback whale are strongly supported. In contrast to other phylogenetic signals incongruent to the species tree, the numbers of TE insertions for the different phylogenetic positions of the gray whale among rorquals are highly similar and make a differentiation between evolutionary scenarios difficult. A ratio of 510:465:444 CHR2 insertions place the gray whale outside a fin, humpback, blue and sei whale clade (510), as sister clade to blue and sei whale (465) or as sister clade to fin and humpback whale (444), respectively (Additional file 1: Figure S4). Hence, this speciation event in the phylogenetic tree appears intuitively as unresolved and in fact a polytomy was only marginally rejected by the KKSC bifurcation test (*p* = 0.0204) [26]. In addition, a plethora of alternative phylogenetic signals of similar strengths illustrate the star-like radiation of Balaenopteridae and Eschrichtiidae. For example, the gray whale shares 433, 374 and 370 CHR2 insertions exclusively with the blue, humpback and fin whale, respectively. With regard to the previously established species tree, these insertions appear to be signals for ILS, however, they can not be considered by the KKSC test [26]. The KKSC test updates the statistical framework introduced by Waddell et al. [28] to test for the significance of conflicting phylogenetic signals from TE insertions to distinguish between ILS and introgression scenarios.

### TE insertion dynamics

To explore the insertion dynamics of CHR2 in baleen whales, we investigated the genetic diversity and the insertion rates across time. We mapped the insertion points of all 91,859 CHR2 insertions on the baleen whale species tree [10] and calculated the frequency of heterozygous insertions on basis of the genotyping information provided by MELT. This allowed us to track how many insertions from each ancestral branch were fixed over time. Not surprisingly, several terminal branches exhibit high rates of heterozygous CHR2 insertions such as the two gray and sei whale populations and the blue whale (Additional file 1: Figure S5). High rates of heterozygous insertions originate also from the ancestral branches that led to the ancestor of gray, fin, humpback, sei and blue whales as well as from the ancestral branch to the fin, humpback and gray whale clade. The genomic heterozygosity of CHR2 insertions was lower in the sei whale branch and the fin and humpback whale clades, branches that exhibit less phylogenetic conflict (Fig. 2).

CHR2 insertion rates were calculated by mapping the insertion numbers on the species tree and using previously estimated divergence times [10] and an average generation time of 24.4 years for extant baleen whales [29]. The estimated insertion rates were relatively stable across the evolutionary lineages and ranged between 0.013–0.138 CHR2 insertions per generation (Additional file 1: Figure S6). The insertion rates at the terminal and shallow branches were relatively low and varied between 0.013 and 0.035. For the ancestral branch to gray, fin, humpback, blue and sei whale a ~ 10-fold increase in insertion rate was observed compared to other branches. The majority of CHR2 insertions that occured on this branch are incongruent to the bifurcating species tree. Repeat landscapes of minke and bowhead whale genome assemblies illustrate the evolution of TE sequences over time, by plotting the frequencies of sequence divergence to the TE consensus sequences. Both whale species show an increase in frequency of low-divergent SINEs (5–10% CpG-adjusted divergence), that could indicate an amplification burst of these elements (Additional file 1: Figure S7). The presence of a similar peak in both species at the same divergence indicate it must have occurred before their divergence at ~ 28 Mya.

## Discussion

Here we have performed the first genome-scale analysis of TE insertions in whales based on next-generation sequencing technology. The included dataset, consisting of 91,859 insertion events across eight baleen whale species, exceeds the dataset size from a previous experimental approach by several magnitudes [16]. Our dataset made it possible to reconstruct the baleen whale evolutionary history and a detailed quantification of phylogenetic conflict.

Many previous studies have attempted to resolve the phylogeny of baleen whales and to clarify the evolutionary origin of the gray whale (family Eschrichtiidae). The gray whale is ecomorphologically derived from the family Balaenopteridae [5, 9] because it is the only bottom-feeding species within a clade of strictly lunge-feeding species [30] leading to confusion about its taxonomic position among baleen whales. Using TEs as virtually homoplasy-free and independent phylogenetic markers overcomes limitations from single-nucleotide based phylogenies [11] and should provide a more detailed understanding about the evolution of baleen whales. Thus, we expected that a detailed analysis of TE insertions would finally settle the baleen whale relationships and also add additional information about the rate of retrotransposition in the slowest evolving mammals.

An evolutionary network analysis together with a detailed analysis of phylogenetically incongruent CHR2 insertions suggests that the speciation of rorquals represents a divergence that might not be entirely dichotomous. This is in spite that the TE based phylogenies were well supported and highly identical to the multi-locus coalescent tree generated from 34,192 sequence based gene trees [10] and a supermatrix tree [7]. However, careful interpretation is warranted given that bootstrap support and posterior probability were designed to assess sampling error of single genes, not genome-scale datasets and might lead to wrong conclusions about the species relationships [31]. Using bootstrap replicates and Bayesian probabilities to infer branch support is common practice, however, well-supported branches might merely be the result of an oversimplified evolutionary model if the dataset is large and the phylogenetic signal is not tree-like. Our in-depth analysis of conflicting synapomorphic TE insertions in baleen whale genomes show that the high statistical support in the phylogenetic trees is based on marginal numeric differences. Unfortunately, methods and models to reconstruct phylogenies from genome-scale multi-locus TE insertion datasets are not as developed as for nucleotide substitutions.

The presence of several equally strong conflicting phylogenetic signals in the CHR2 dataset can be caused by a) insufficient character sampling leading to an unresolved divergence (soft polytomy), b) near-instantaneous speciation and subsequent incomplete lineage sorting (ILS), or c) speciation under genetic exchange. Given the data presented here, it is highly unlikely that the divergence of the gray whale and its sister lineages represent a soft polytomy (a), as our extensive dataset of 91,859 CHR2 insertions is distributed across the near complete 2.3 Gb genome sequence of baleen whales and each node in the phylogeny is supported by several hundred insertions (Fig. 2b). In addition, a confounding effect from incorrect phylogenetic signal is marginal because SINE insertions are virtually free from homoplasy.

ILS (b) is the persistence of ancient polymorphisms across speciation events and has been observed in several TE-based phylogenomic studies [32–34], including a study investigating baleen whale relationships [16]. Several factors, such as a rapid radiation, large or expanding ancestral effective population sizes (N_e_) and consequently a slow evolutionary fixation rate favor the occurrence of ILS [33]. The gray whale and the ancestors of the blue- plus sei whales and fin- plus humpback whales rapidly diverged from each other within less than one million years, as is evident from the star-like phylogenetic network (Fig. 2a) and previous divergence time estimates [10, 35]. In addition, a large ancestral N_e_ is suggested by the high number of species-tree incongruent CHR2 insertions and the large fraction of evolutionary old and still unfixed, heterozygous insertions that integrated on the ancestral branches with the highest degree of ILS (Additional file 1: Figure S5, and S6). The genome-wide analysis of CHR2 insertion thus strongly indicates that the ancestral rorqual population exhibited large population sizes and radiated rapidly. Also, explicit modeling of the demographic histories of baleen whales based on genomic data indicates large ancestral population sizes of whales [10]. However, these estimates do not reach back enough in time to cover the timeframe of the radiation.

Whales are the largest living animals and known for their slow physiological and evolutionary rate [36]. They exhibit the slowest nucleotide substitution rate among mammals, estimated to be 10 times slower than among primates [37]. Our estimates indicate that the rate of SINE insertions is about 50% slower than in humans, for which a mean rate of 0.046 Alu insertions per generation per genome was estimated [38]. However, we also observe a 10-fold increased CHR2 insertion rate on the branch to the fin, humpback, gray, blue and sei whale clade. Similar strong fluctuations in SINE insertion rates across evolutionary time, like estimated within baleen whales, were also reported for great apes [20].

Finally, a potential third cause for a conflicting phylogenetic signal (c) is that the emerging whale species might have exchanged genetic material for a long time because vicariance is more difficult to maintain in the marine than in the terrestrial environment. Hence, also speciation with genetic exchange of baleen whales might have caused trans-species polymorphisms [10, 39]. Whether the resulting genomic mosaicism is a result of speciation with genetic exchange or from ILS is however not possible to determine [40] and both processes are plausible for baleen whales. Either process or a combination of both could have created the observed phylogenetic signals that are incompatible with a strictly bifurcating tree. More detailed investigation of these processes require new methods that examine patterns of phylogenetic signals from TE insertions with respect to speciation processes and gene flow.

## Conclusions

This study demonstrates the suitability of WGS datasets to infer TE insertions, one of the largest contributor to genomic variation in mammals [41]. Thus, TE insertions are a highly valuable source for comparative genomics and for reconstructing phylogenies. In line with the first application of TE-based phylogeny of baleen whales [16] and a recent nucleotide-based study [10], the radiation of rorquals sensu lato appears to represent a hard polytomy when depicted as a phylogenetic tree because alternative phylogenetic scenarios are equally well supported. Therefore, a better representation of the rorquals’ evolutionary history would be to represent the divergences in a phylogenetic network [10], allowing for the incorporation of ILS and genetic exchange between species as horizontal reticulations. We anticipate that a population-wide sampling of baleen whales might illuminate the divergence processes in more detail.

## Materials and methods

### WGS mapping

Whole-genome sequencing data from ref. 10 plus additional samples of two gray whales and a fin whale [42, 43] were quality-checked with FastQC (https://www.bioinformatics.babraham.ac.uk/projects/fastqc/), trimmed if necessary with Trimmomatic [44] and mapped to the bowhead whale genome with BWA [45] (Additional file 1: Table S1). The bowhead whale (*Balaena mysticetus*) genome assembly [13] was chosen for reference mapping over the more continuous minke whale genome because it is a natural outgroup to the rorqual species and thus eliminates TE detection bias between samples [23].

### TE detection

The Mobile Element Locator Tool (MELT) [19] was run in the Split mode on all scaffolds larger than 100 kb. A consensus file for TE detection was created according to the MELT manual. We chose the general consensus sequence of the CHR2 SINE family, that was active during the evolution of Cetacea [46]. Seven different subfamilies of CHR2 have been described for cetaceans [47], that contain indels compared to the general CHR2 consensus sequence. Using the full length general consensus of CHR2 [14] and allowing for 10% mismatches makes a broader detection of CHR2 insertions in MELT possible. To annotate all copies of the CHR SINE family elements in the bowhead whale genome, the genome sequence was repeat-masked (http://www.repeatmasker.org/) with the Cetartiodactyla repeat library. BEDOPS [48] converted the RepeatMasker output into BED format.

### Simulation and sensitivity analysis

Prior to TE calling, we performed a sensitivity and specificity analysis using our custom-made TE calling assessment pipeline ESAT (Element Simulation Analysis Tool) using sequences and parameters matching our whale dataset. We selected the longest scaffold (5 Mb) from the bowhead whale assembly to serve as a sample genome for our sensitivity analysis. We randomly integrated 200 CHR2 SINEs in the sample genome sequence and simulated paired-end Illumina reads from the resulting sequence with SimSeq (https://github.com/jstjohn/SimSeq) at sequencing coverage levels ranging from 1 to 30 X coverage. For read simulation we generated an error-profile typical for our whale resequencing datasets. Reads were mapped to the sample genome with BWA [45] as described above and MELT was used to call the CHR2 SINE insertions from our simulated genome. We generated 10 replicates per simulation. To analyze the performance of MELT, we assessed if the detected non-reference TE insertions matched the simulated TE locations using BEDtools [49]. The detection rate (DETR) reflects the sensitivity of MELT to successfully identify a TE insertion. True positive rate (TPR), false positive rate (FPR) and false negative rates (FNR) were calculated from the detected TEs to estimate MELT’s accuracy on the whale dataset. Finally, the proportion of correctly genotyped insertions among the detected variants was recorded. We made ESAT publicly available on https://github.com/crueckle/ESAT.

### Phylogenomic analysis

Orthologous TE insertion calls across the taxon sampling were identified using the GroupAnalysis and Genotype algorithms in MELT. TE insertion calls passing internal MELT filters were extracted with bcftools filter (www.htslib.org). A NEXUS-formatted presence absence matrix of orthologous TE insertions was created with a modified version of vcf2phylip [50]. Phylogenies were reconstructed using Neighbor-Joining and Dollo Parsimony in PAUP* [51]. Under Dollo Parsimony, only character state changes from absence to presence (0 to 1) are allowed, thus matching the evolutionary model of TE insertions. Heuristic tree search was conducted with random addition of sequences and 100 repetitions using Tree Bisection and Reconnection (TBR) as branch swap algorithms. Bootstrap support values were calculated from 1000 replicates. Likelihood scores for each tree were calculated using the ‘lscores’ command. A Bayesian inference tree was calculated in MrBayes v.3.2.6 [52] using “irreversible” character type (ctype irreversible:all) with 10e7 generations and sampling every 1000th generations, 25% of the samples were discarded as burn-in. Principal component analysis (PCA) for the filtered CHR2 datasets were conducted with the SNPRelate package for R. Phylogenetic median joining networks were generated in SplitsTree4 [53]. The intersection diagram was created with UpSetR [54]. For gray and sei whales, only TE insertions present in all individuals of the respective species were considered.

### Insertion rates

Per-branch insertion rates were calculated from the number of CHR2 insertions that we had mapped to the species tree from ref. 10. This tree was used because it is the best available bifurcating representation of the baleen whales evolutionary history and is congruent with other recent studies on baleen whale phylogeny [7]. Species-tree incongruent CHR2 insertions were assumed to be the result of ILS and accordingly mapped to the most recent ancestral branch leading to the affected species. The insertion rate was calculated by the equation *μ* = *η*_*CHR2*_^∗^*b*/24.4 with *n*_*CHR*2_ for the number of CHR2 insertions and *b* as the branch length in years. The mean generation time of 24.4 years was calculated for from recent generation time estimates of the studied species [29].

## Additional files

Additional file 1: **Table S1.** List of samples with accession numbers and sequencing properties. **Figure S1.** Simulation results for CHR2 detection with MELT at varying depth of coverage using dataset specific parameters. **Figure S2** Frequency of filters applied by MELT to exclude low-quality CHR2 calls. **Figure S3** Phylogenetic trees of baleen whales reconstructed with CHR2 insertions. A) Dollo-Parsimony tree reconstructed in PAUP*. Asteriks indicate 100 % bootstrap support (500 replicates), lower bootstrap support is given as numbers. B) Bayesian inference tree with posterior probability given for nodes. **Figure S4** Three alternative relationships in the rorqual radiation and the number of CHR2 insertion that support them. **Figure S5** Phylogenetic tree of rorquals with frequency of heterozygous insertions per branch. **Figure S6** CHR2 insertion rates per generation. **Figure S7** Repeat landscapes of minke whale and bowhead whale based on available assemblies. (PDF 475 kb)

Additional file 2: **Data S1**: VCF file with filtered CHR2 variants in baleen whales called by MELT. (ZIP 10547 kb)

Additional file 3: **Data S2**: NEXUS file with the presence-absence matrix of CHR2 insertions in baleen whales encoded as 1 (presence) and 0 (absence). (ZIP 159 kb)

## Abbreviations

BI: Bayesian inference
CI: Consistency index
DETR: Detection rate
ESAT: Element simulation analysis tool
FNR: False negative rate
FPR: False positive rate
ILS: Incomplete lineage sorting
MELT: Mobile element locator tool
Mya: Million years ago
N_e_: Effective population size
NJ: Neighbor-Joining
PCA: Principal component analysis
SNV: Single nucleotide variant
TBR: Tree bisection and reconnection
TE: Transposable element
TPR: True positive rate
WGS: Whole genome sequencing
