# Supplementary material for: Retrophylogenomics in rorquals indicate large ancestral population sizes and a rapid radiation
Source: Mob DNA. 2019 Jan 21;10:5. doi: 10.1186/s13100-018-0143-2 (PMC6340175; doi:10.1186/s13100-018-0143-2)
Supplement: Supplementary file 1 — Table S1. List of samples with accession numbers and sequencing properties. Figure S1. Simulation results for CHR2 detection with MELT at varying depth of coverage using dataset specific parameters. Figure S2 Frequency of filters applied by MELT to exclude low-quality CHR2 calls. Figure S3 Phylogenetic trees of baleen whales reconstructed with CHR2 insertions. A) Dollo-Parsimony tree reconstructed in PAUP*. Asteriks indicate 100 % bootstrap support (500 replicates), lower bootstrap support is given as numbers. B) Bayesian inference tree with posterior probability given for nodes. Figure S4 Three alternative relationships in the rorqual radiation and the number of CHR2 insertion that support them. Figure S5 Phylogenetic tree of rorquals with frequency of heterozygous insertions per branch. Figure S6 CHR2 insertion rates per generation. Figure S7 Repeat landscapes of minke whale and bowhead whale based on available assemblies. (PDF 475 kb) [file 13100_2018_143_MOESM1_ESM.pdf]

Retrophylogenomics in rorquals indicate large ancestral population sizes and rapid radiation

Supplementary Material

Fritjof Lammers, Moritz Blumer, Cornelia Rücklé, Maria A. Nilsson

Supplementary Tables

Table S 1. List of samples with accession numbers and sequencing properties

Species	ID	Accession-ID	Reference	Insert Size	Coverage
NA right whale	EgI0	SRR5665640	Arnason et al. 2018	470	10.69x
Sei whale A	Bbo01	SRR5665645	Arnason et al. 2018	482	10.36x
Sei whale B	Bbo02	SRR5665646	Arnason et al. 2018	482	10.27x
Blue whale	Bmu00	SRR5665644	Arnason et al. 2018	293	30.68x
Humpback whale	Mno00	SRR5665639	Arnason et al. 2018	470	27.88x
Fin whale	Bph03	SRR5665643	Arnason et al. 2018	294	13.99x
Minke whale	Bac00	SRR896642	Yim et. al 2014	472	7.73x
Gray whale (eastern) A	Ero01	SRR5665641	Arnason et al. 2018	284	17.60x
Gray whale (eastern) B	Ero02	SRR5665642	Arnason et al. 2018	284	22.29x
Gray whale (western) A	Ero03	SRR5495108	DeWoody et. al. 2017	497	29.24x
Gray whale (western) B	Ero04	SRR5495104	DeWoody et. al. 2017	471	26.65x

Supplementary Figures

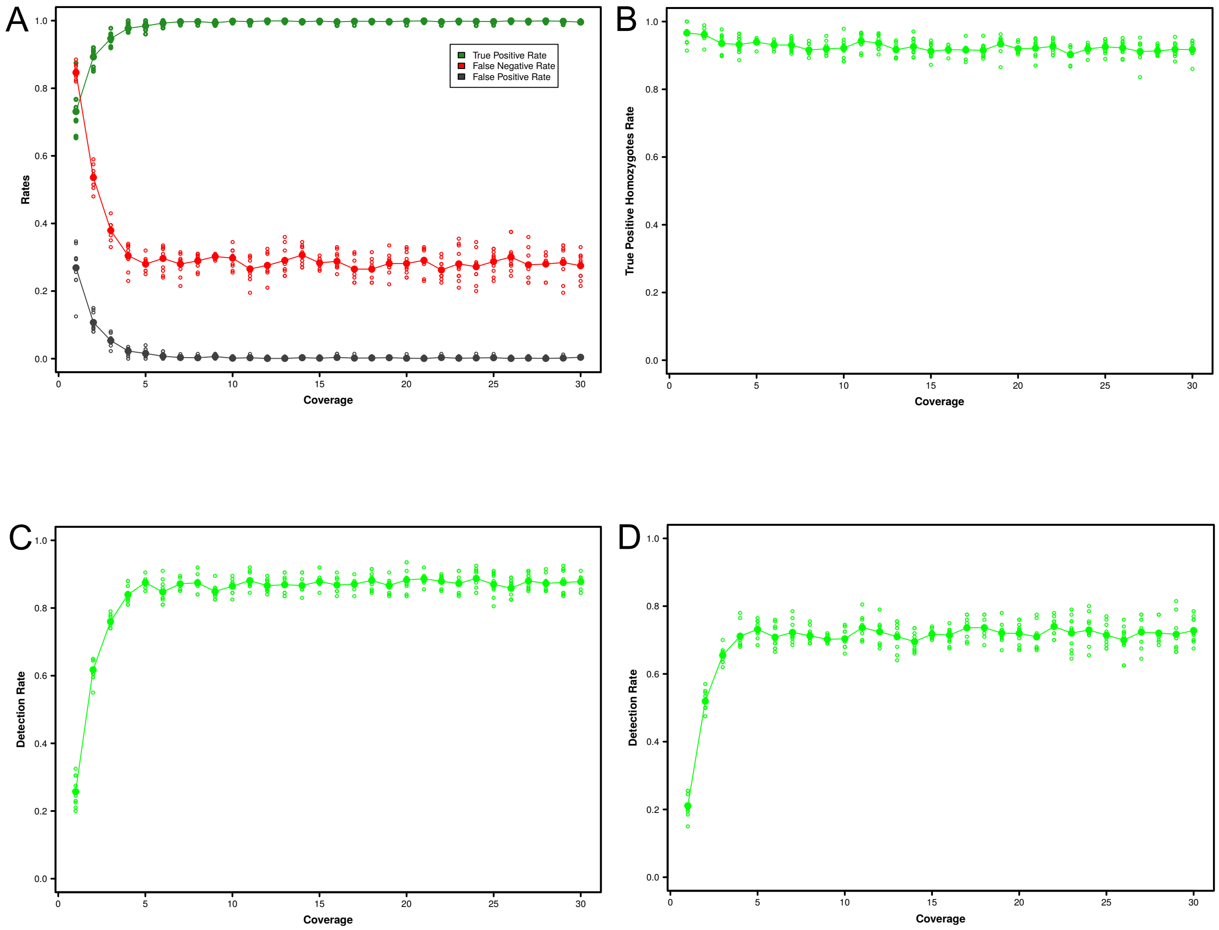

Figure S 1. Simulation results for CHR2 detection with MELT at varying depth of coverage using dataset specific parameters. A) Accuracy rates for detected CHR2 calls at varying coverages. B) Genotyping accuracy estimated from CHR2 insertions correctly called homozygous. C) MELT detection rate for the unfiltered and D) filtered datasets using MELTs internal filtering.

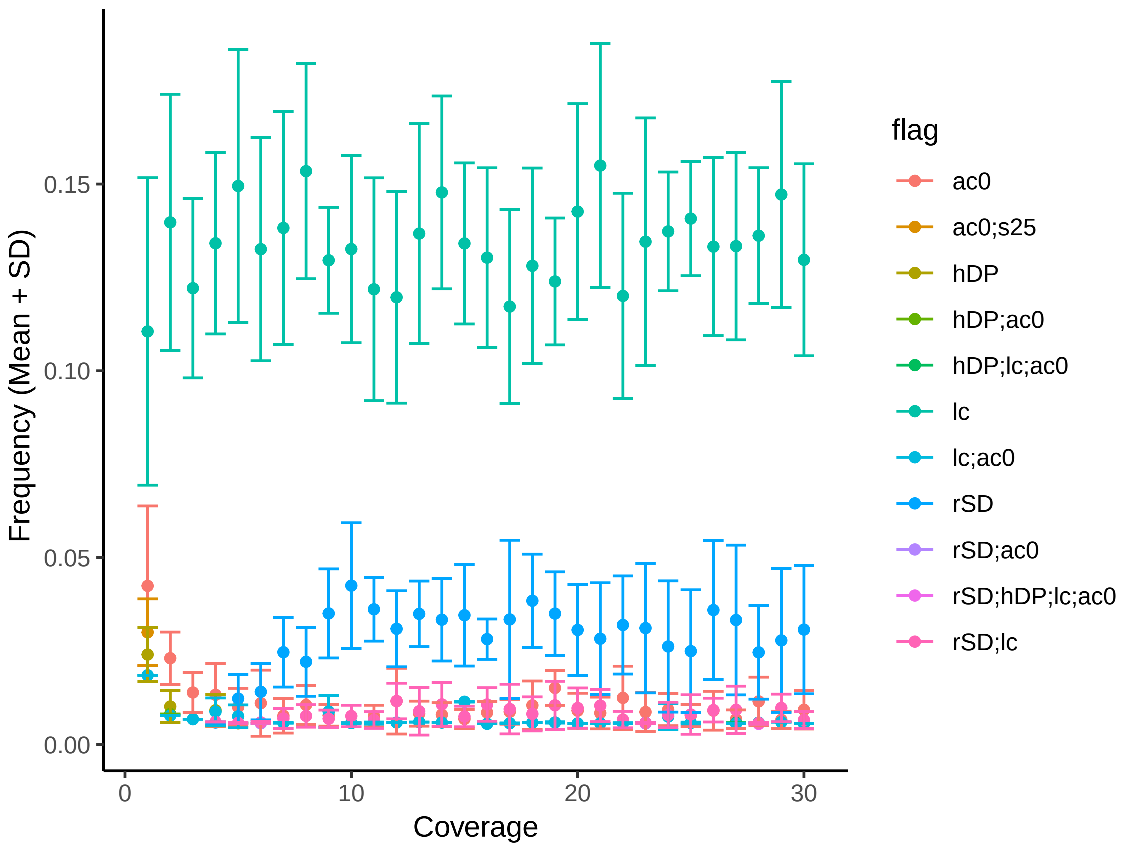

Figure S 2. Frequency of filters applied by MELT to exclude low-quality CHR2 calls. Most calls were excluded for proximity to low-complexity regions (lc). The other filter flags indicate sites without genotyped allele (ac0), sites that are not called in at least 25% of individuals (s25), sites with a high degree of discordant read pair evidence (hDP) and sites having unbalanced evidence on the 5' and 3' flanks of the insertion site (rSD). The description of filter flags are according to the MELT Documentation (http://melt.igs.umaryland.edu/manual.php).

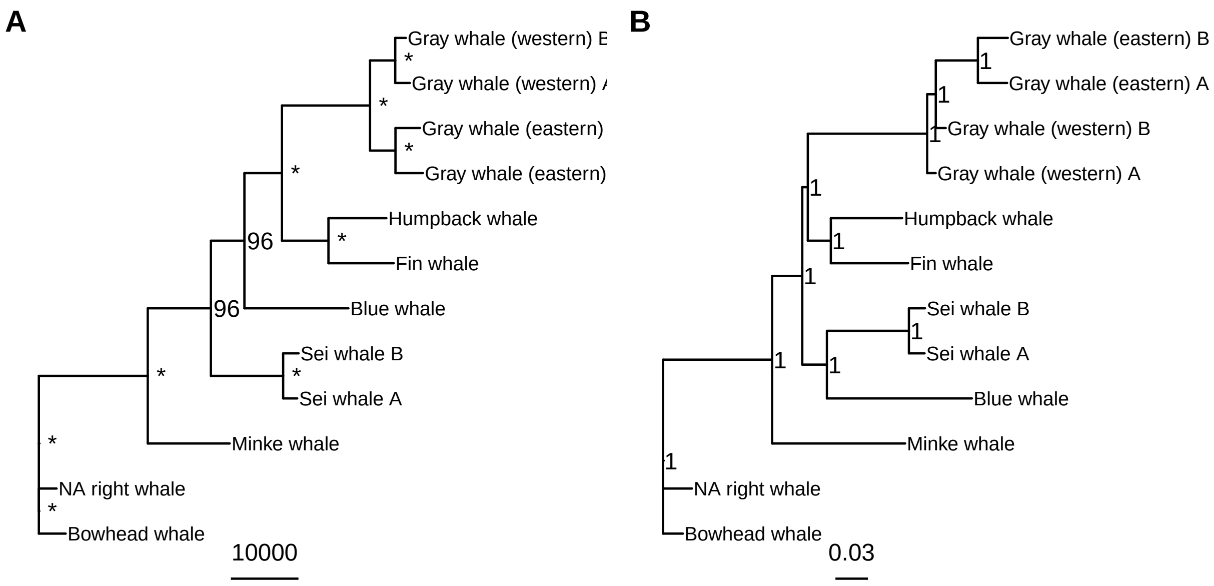

Figure S 3. Phylogenetic trees of baleen whales reconstructed with CHR2 insertions. A) Dollo-Parsimony tree reconstructed in PAUP*. Asteriks indicate 100 % bootstrap support (500 replicates), lower bootstrap support is given as numbers. B) Bayesian inference tree with posterior probability given for nodes.

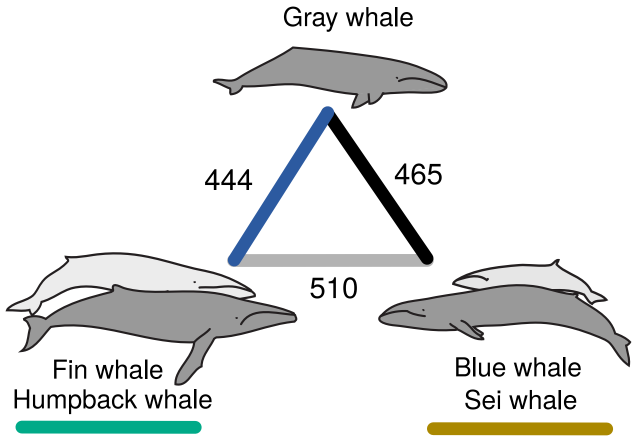

Figure S 4. Three alternative relationships in the rorqual radiation and the number of CHR2 insertion that support them. The KKSC test marginally rejects polytomy at p=0.02 (bifurcation test). The colors represent the edges in the network and signal quantification in Figure 2.

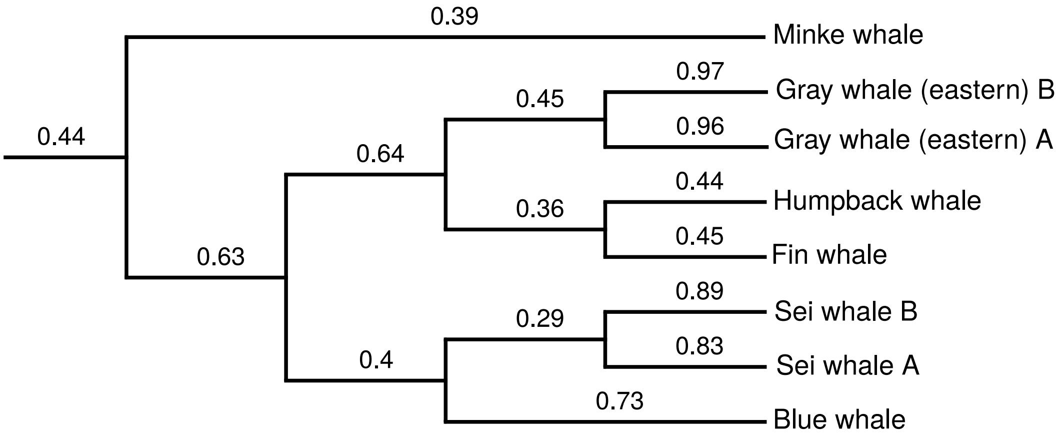

Figure S 5. Phylogenetic tree of rorquals with frequency of heterozygous insertions per branch. Heterzygous insertion frequency are calculated among extant species and mapped to the branches. Gray whale heterozygosity rates were calculated for each population (eastern and western Pacific).

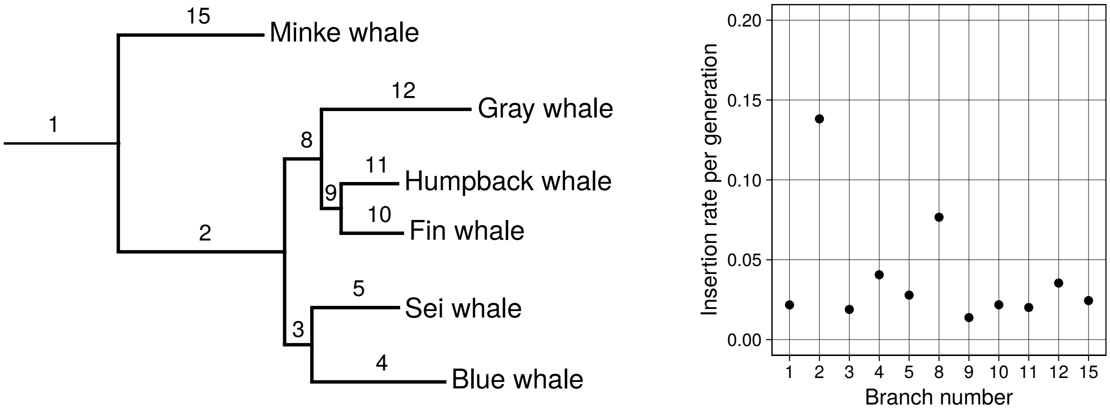

Figure S 6. CHR2 insertion rates per generation. Divergence times are taken from reference no. 10. The average insertion rate of baleen whales was calculated to be 24.4 years.

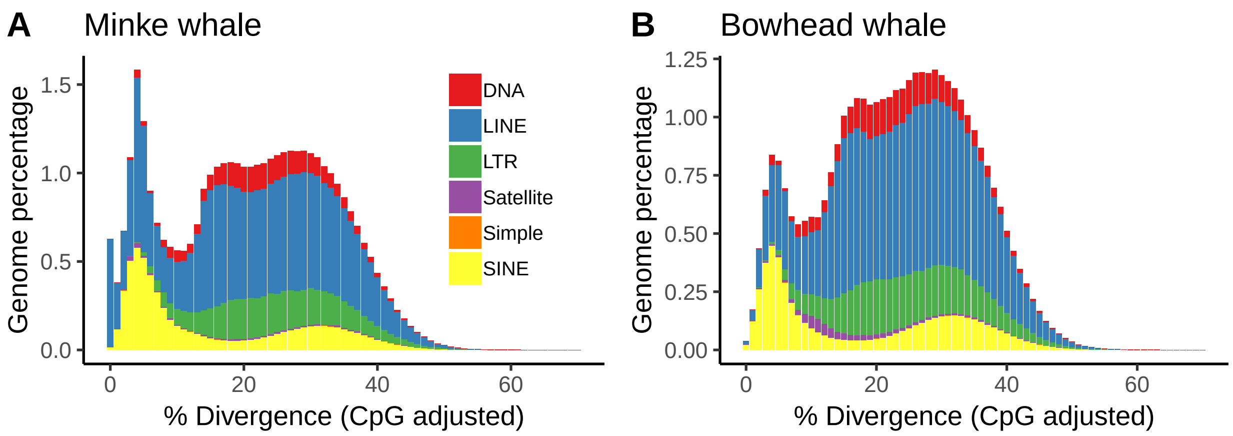

Figure S 7. Repeat landscapes of minke whale and bowhead whale based on available assemblies. The diagrams show the genome percent of the major repeat types depending on how much they have diverged from the consensus sequences.
